# Advances in Intestinal Barrier Preservation and Restoration in the Allogeneic Hematopoietic Cell Transplantation Setting

**DOI:** 10.3390/jcm10112508

**Published:** 2021-06-06

**Authors:** Martyna Tyszka, Jarosław Biliński, Grzegorz Władysław Basak

**Affiliations:** Department of Hematology, Transplantation and Internal Medicine, Medical University of Warsaw, 02-097 Warsaw, Poland; jaroslaw.bilinski@gmail.com (J.B.); grzegorz.basak@wum.edu.pl (G.W.B.)

**Keywords:** intestinal barrier, gut permeability, microbiome, allogeneic hematopoietic cell transplantation, graft-versus-host disease

## Abstract

The intestinal barrier consists of an epithelial lining covered with specialized mucus inhabited by intestinal microbiota. An intact gut barrier ensures a resistance to bacteria and toxins translocation. On the other hand, gut permeability allows the absorption of essential nutrients, fluids and ions. This balance is achieved only by the complex structure and functional characteristics of the intestinal barrier. Allogenic hematopoietic cell transplantation remains the only curative treatment for many hematological diseases, but its application is limited because of possible transplant-related mortality mainly due to graft-versus-host disease and infectious complications. The intestinal barrier has been extensively studied in recent years as the primary site of graft-versus-host disease initiation and propagation. In the present review, we focused on the physiological structure and function of the gut barrier and the evidence of how the disruption of the gut barrier and increased intestinal permeability affects transplant recipients. Finally, therapeutic strategies aiming at intestinal barrier protection with a special focus on microbiome preservation and restoration in the allogenic hematopoietic cell transplantation setting are discussed.

## 1. Introduction

In the past few decades, many studies investigating the connection between intestinal barrier function and allogenic hematopoietic cell transplantation (allo-HCT) outcomes, especially its major complication—graft-versus-host disease (GVHD), were conducted. Evidence on the intestinal barrier as the primary site of GVHD development is piling up, although we still do not fully understand the cellular and molecular mechanisms of GVHD initiation and propagation. Intestinal barrier disruption caused by conditioning promotes pathogen-associated molecular pattern (PAMP) translocation to the lamina propria and activation of the proinflammatory pathways at the basis of GVHD initiation [1]. The protective function of a proper intestinal barrier and healthy microbiota compartment was investigated in relation to GVHD incidence. In a multicenter study, preserved microbiome diversity assessed with 16S rRNA sequencing at the time of hematopoietic cell engraftment was associated with significantly lower mortality [2]. Every single research paper leading to a better understanding of the relationship between intestinal barrier disruption and outcomes of allo-HCT is bringing us closer to the more effective GVHD prophylaxis and treatment. The overall goal of this review is to provide a comprehensive analysis of the intestinal barrier function in patients subjected to allo-HCT. In this article, we focused on the physiological structure and function of the intestinal barrier, the evidence on disruptions of the gut barrier and increased intestinal permeability in allo-HCT recipients and, finally, therapeutic strategies for intestinal barrier preservation and restoration.

### 1.1. Intestinal Barrier Anatomy and Physiology

**Figure 1:**
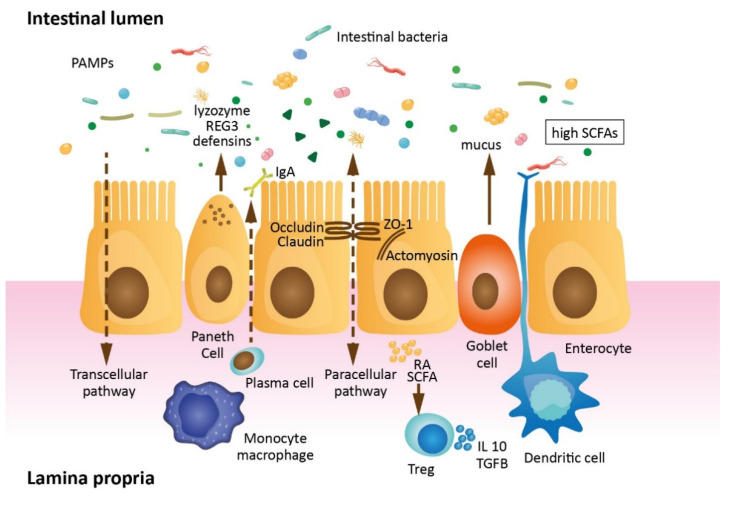
Intestinal barrier. Apically placed tight junctions formed by transmembrane proteins (e.g., claudins and occludin) and peripheral scaffolding proteins (e.g., zonula occludens; ZO-1) seal the paracellular space between enterocytes and form the barrier limiting the intestinal microorganisms and pathogenic molecules (PAMPs) passage and, on the other hand, enabling the paracellular absorption of the essential nutrients and ions, depending on the size and charge. The lamina propria is inhabited by the cells of the immune system, e.g., T cells, plasma cells, macrophages and dendritic cells. Tregs modulate the immune system into a tolerance to self-antigens and chronic stimuli by the secretion of Il-10 and TGF-beta and suppression of effector T cells. The intestinal barrier is maintained by the SCFAs produced by the commensal bacteria and several protective molecules, i.e., lysozyme, defensins, REG3 and IgA, secreted by specialized cells of the intestinal lining. ZO-1, zonula occludens-1, DAMPs, damage-associated molecular patterns, PAMPs, pathogen-associated molecular patterns, IL-10. Interleukin 10, TGF-beta, transforming growth factor-beta, SCFAs, short-chain fatty acids, REG3, regenerating islet-derived protein 3 and IgA, Immunoglobulin A.

The intestinal barrier is formed by consecutive layers, including intestinal epithelial cells (IECs) covered with mucus inhabited by the intestinal microflora and innermost lamina propria (Figure 1). Enterocyte membranes are interconnected with each other with junctional complexes: tight junctions (TJs), adherens junctions (AJs) and desmosomes. Apically placed TJs are formed by transmembrane and peripheral scaffolding proteins. AJs consisting of transmembrane protein E-cadherin connected to the intracellular skeleton are critical for TJ assembly and cell-to-cell adhesion. Desmosomes built by desmoglein and desmocollin are believed to strengthen the intercellular cohesion [3,4,5,6]. The mucus primarily secreted by goblet cells forms an inhomogeneous structure. The inner layer is virtually sterile, assists nutrient absorption, ensures epithelial hydration and protects the epithelial cells from luminal shear stress and digestive tract enzymes. The outer layer of mucus prevents bacteria from adhering to the intestinal epithelium and contains several antimicrobial agents, such as secretory immunoglobulin A, defensins, ribonucleases and lysozymes [7].

Commensal intestinal microflora (microbiota) occupies the outer layer of the mucus. A healthy individual is host to around 10^14^ microbial organisms: over 2000 species of bacteria (most of which are from the Firmicutes, Bacteroidetes, Actinobacteria and Proteobacteria phylum), as well as viruses and fungi, depending on age, gender, body mass index, mode of birth and diet [8]. The intestinal microbiota interplays with its host in several ways, by direct interactions with the cells of the intestinal barrier or via various microbial metabolites. Commensal bacteria prevent pathogen growths by niche occupation, nutrient competition and the production of several bacteriocins. They also promote the secretion of antimicrobial peptides (AMPs), including defensins and cathelicidins via the activation of pattern recognition receptors (Toll-like receptors and Nod-like receptors) in IECs [9,10]. Another important role of the intestinal bacteria is the fermentation of undigested food residues to short-chain fatty acids (SCFAs), which are the source of energy for colonocytes. SCFAs maintain intestinal endothelium continuity and affect cell morphology by increasing the number of intestinal villi and improving the junctional integrity. They maintain the balance between pro- and anti-inflammatory responses through a set of free fatty acid receptors (FFARs) and the induction of regulatory T cells (Treg cells) expressing transcription factor Foxp3 by the inhibition of the histone deacetylase enzyme [11,12,13]. Another bacterial metabolite, indoxyl sulfate (IS), which is a derivate of indole, the main tryptophan metabolite, is known to preserve a mixed commensal microbiome by exerting bacteriostatic effects on Gram-negative bacilli and cocci [14] and providing colonization resistance to *Candida albicans* [15]. It also protects the epithelial cell barrier by simultaneously decreasing the expression of the proinflammatory chemokine IL-8 and TNF-α-mediated activation of NF-κB and increasing the expression of the anti-inflammatory cytokine IL-10 and type I interferon (IFN1) response [16]. 

Intestinal stem cells residing at the bottom of the crypts of Lieberkühn allow barrier renewals approximately every 3–5 days. As the fully matured cells undergo apoptosis and are being shed into the intestinal lumen, stem cells proliferate and differentiate into enterocytes, goblet cells, Paneth cells and enteroendocrine cells. Below the epithelial lining lies the lamina propria, populated by cells of the enteric nervous system and immune cells [3,4].

### 1.2. Gut Barrier Permeability

Intestinal permeability is a function that allows the selective transfer of defined molecules across the intestinal wall and nutrient absorption. In healthy individuals, the permeability for electrolytes and nutrients is divided into two main pathways. The paracellular route ensures that 85% of the total influx of molecules is regulated by TJs, and the transcellular route allows solute transportation across the enterocyte membrane. The pore size in the TJ is the primary determinant of mucosal permeability in the presence of intact epithelium transport pathways [17]. 

A barrier function regulation results from interactions between the microbiota, epithelial cells, immune system and ENS and can be altered by gut microflora modifications, mucus layer alterations and epithelial damage [7].

## 2. Impact of allo-HCT Procedure on Gut Barrier Permeability (Mechanisms and Summary of Studies)

Oral mucositis affects 60–100% of HCT recipients when myeloablative conditioning regimens are used [18]. It is hard to overlook and easy to evaluate with the World Health Organization (WHO) oral mucositis scores. The damage to the gastrointestinal tract is less understood, even though signs and symptoms from the GI tract such as abdominal pain, nausea, vomiting and diarrhea occur often after allo-HCT. The intestinal permeability is most commonly measured by the difference in intestinal barrier passage and fractional urinary excretion of various ingested probes of different sizes. A few studies evaluating intestinal permeability assessed by a 51CrEDTA absorption test or sugar absorption test (SAT) [19,20,21,22,23] have shown that the gut permeability was raised significantly after the start of conditioning, and it preceded any clinical signs of mucosal damage. The clinical gastrointestinal toxicity and oral mucositis grade did not strictly correspond with the intestinal injury. These results suggest that it may be misleading to estimate the extent of damage to the GI tract based on the grade of the oral mucositis at the posttransplant period in the same patient. 

Another way to investigate the gut barrier disruption is the evaluation of the serum levels of various biomarkers. Those can be divided into two categories: bacteria-related molecules like lipopolysaccharide (LPS) [24] and circulating endotoxin core antibodies (EndoCAb) [25] or direct intestinal barrier damage markers like citrulline produced by small intestinal enterocytes [26,27], fatty acid-binding proteins (FABPs) [28] or tight junctions proteins—claudins [29]. A significant decrease in the serum concentrations of citrulline after HCT was seen in a study of 32 patients receiving myeloablative therapy. In a subgroup of 12 patients, a lower serum citrulline was correlated with the onset of oral mucositis and increased intestinal permeability measured with SAT. Serum citrulline appears to be a simple test to implement in studies on the gut barrier after HCT [26]. In another study, high serum levels of IL-8, lipopolysaccharide-binding protein (LBP) and C-Reactive Protein (CRP) corresponded with citrulline concentration nadir and increased intestinal permeability measured with SAT after allo-HCT. The authors suggested that the observed systemic inflammatory response after HCT began with intestinal barrier damage [30]. In a study focusing on the severity of GVHD and intestinal toxicity assessed by a 51Cr-EDTA absorption test, it was confirmed that patients with mild acute GVHD had better-preserved gut barrier function and exhibited less-pronounced gut toxicity in comparison with patients with more severe acute GVHD. In this study, the intestinal barrier permeability and intestinal toxicity were the only factors significantly predicting acute GVHD severity, underlining the importance of gut barrier disruption for the development of GVHD [23].

## 3. Gut Barrier Preservation and Restoration in allo-HCT

**Figure 2:**
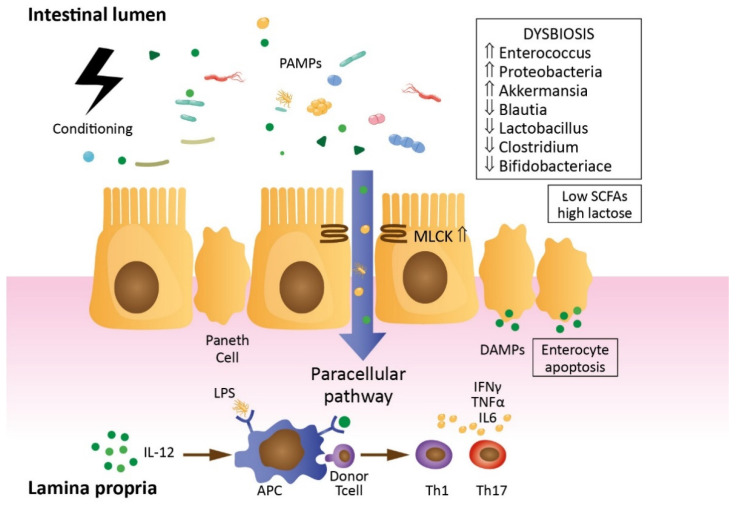
Intestinal barrier disruption after allo-HCT. Pretransplant conditioning results in enterocyte and Paneth cells apoptosis, leading to lower lactase and higher lactose concentrations and dysbiosis with the *Enterococcus* genus domination associated with lower SCFA concentrations. An increased MLCK expression results in loosening of the tight junctions and PAMPs translocation into the lamina propria. PAMPs (e.g., LPS) and DAMPs from damaged cells activate APCs. It results in donor T-cell activation, proinflammatory cytokines, i.e., IFN-γ, TNF-α, IL-5 release and GVHD. SCFAs, short-chain fatty acids, MLCK, myosin light chain kinase, PAMPs, pathogen-associated molecular patterns, LPS, lipopolysaccharide, DAMPs, damage-associated molecular patterns, APC, antigen-presenting cell, IFN-γ, interferon gamma, TNF-α, tumor necrosis factor alpha, IL-5, interleukin 5 and GVHD, graft-versus-host disease.

GVHD remains the major complication of allo-HCT, worsening the outcomes of its recipients. Previous studies of inflammatory pathways involved in GVHD development in animals showed that the gastrointestinal tract is not only the major target organ but, also, the place of the GVHD initiation and propagation [31,32,33,34,35]. Studies have shown that intestinal barrier damage during transplantation results in increased permeability and, hence, increased intestinal PAMPs translocation and the production of proinflammatory cytokines, which causes a “cytokine storm”, along with the activation of alloreactive T lymphocytes [36] (Figure 2). The means of GVHD prevention are the enhancement of the GI mucosal barrier structure (including microbiome) and restoration of the barrier function altered by the conditioning.

### 3.1. Vitamin A

Recently, free serum vitamin A levels were tested in a cohort study of 114 consecutive pediatric patients undergoing allo-HCT. Lower vitamin A levels after transplantation were associated with a higher rate of GI GVHD, along with TRM at 1 year [37]. In the gut, dendritic cells metabolize vitamin A into retinoic acid (RA). RA promotes the differentiation of Foxp3+ regulatory T cells and immunoglobulin A antibody-secreting cells [38] and the secretion of interleukin 22 (IL-22), which supports epithelial cell proliferation, TJ functions and mucus production by goblet cells [39]. Studies to evaluate the benefits of vitamin A supplementation on the intestinal barrier integrity in allo-HCT are required.

### 3.2. MLCK Inhibition

In vitro and animal studies have identified myosin light-chain kinase (MLCK210) as a major regulator of tight junction functions. An increase in MLCK210 expression was associated with impaired barrier functions in lung injury models, and a treatment with the MLCK inhibitor resulted in protection against lung injury [40,41]. Recently, Nalle et al. investigated MLCK210 in the animal GVHD model. MLCK210-deficient mice did not experience epithelial barrier loss and showed less GVHD propagation, marked by reduced histopathology, fewer CD8+ effector T cells in the gut and improved overall survival [42]. All this data place MLCK as a target for a therapeutical approach in GVHD treatment beyond immunosuppression. 

### 3.3. Indole Derivatives

As mentioned above, indole derivates are exerting protective functions towards the intestinal epithelial barrier. It was shown that the treatment with indole-3-carboxaldehyde (ICA) limited the epithelial damage associated with conditioning, bacterial translocation and inflammation, reducing GVHD-related mortality in a murine model [43]. Further studies on ICA as the prophylaxis against GVHD are needed.

### 3.4. Alloantigen Presentation Inhibition in the Gut via IL-12 Neutralization

The initiation of GVHD starts with the interactions between recipient antigen-presenting cells (APCs) and donor T cells. It is well-known that not only hematopoietic host APCs but, also, nonhematopoietic (“nonprofessional”) APCs like fibroblasts or endothelial cells can also initiate MHC-II-dependent responses [44,45]. A recent paper from Koyama et al. showed that intestinal microflora stimulates MHC-II expression in intestinal epithelial cells and that this process is further enhanced after the start of conditioning therapy. It was also shown that MHC-II presentation by IECs is necessary for CD4+ T-cell-mediated GI GVHD. The pretransplant neutralization of IL-12 prevented the MCH-II expression on ICSs and mitigated GI GVHD in mice [46]. The IL-12 blocking antibody is already used in humans for the treatment of inflammatory bowel disease, plaque psoriasis and psoriatic arthritis and has shown promising results in blocking Th1/Th17 responses after allo-HCT, as demonstrated in a small trial that was not powered for clinical benefit [47]. 

### 3.5. Intestinal REG3

Regenerating islet-derived protein 3 (REG3) is an antibacterial protein essential for epithelial barrier preservation by the protection of intestinal stem cells, promotion of epithelial proliferation and reduction of crypt inflammation and apoptosis following the tissue damage [48,49,50]. An elevated REG3α blood level associated with the loss of Paneth cells is an established marker of GI GVHD and predictor of TRM [51,52]. Increased intestinal REG3 was observed after IL-22 [48], lipopolysaccharide [53] or propionate [50] administration. Strategies aiming at the restoration of REG3-mediated epithelial barrier homeostasis may offer a novel GVHD prophylaxis and treatment modality.

### 3.6. Butyrate

Short-chain fatty acids are the product of the bacterial fermentation of dietary fibers. It was shown in vitro that reduced butyrate in IECs after allo-HCT resulted in decreased histone acetylation, which was restored after the local administration of exogenous butyrate. Butyrate supplementation improved the IEC junctional integrity, decreased apoptosis and mitigated GVHD in an animal model [54]. Patients who developed chronic GVHD had significantly lower concentrations of butyrate late after HCT (day +100) compared to those without such complications [55]. Another study showed that butyrogenic bacteria abundance at the time of engraftment was a protective factor against viral respiratory tract infections in the posttransplant period [56]. Finally, data from a single-center pilot study that confirmed the correlation between fecal butyrate and indole concentrations with microbiota diversity after HCT also reported that patients with lower fecal metabolites levels were at a higher risk of contracting bloodstream infections within 30 days after transplantation [57]. 

### 3.7. Prebiotic Use

A recent prospective study on prebiotic effects on intestinal barrier preservation was conducted, with promising results. Resistant starch dishes and a commercially available prebiotic mixture were administered to 49 patients from before conditioning to day 28 after allo-HSCT. In comparison to the historical data, the prebiotic intake alleviated the mucosal injury and reduced the risk of aGVHD. The prebiotic use also resulted in the preservation of microbiota diversity and butyrogenic bacteria prevalence, with a high post-transplantation fecal butyrate concentration [58]. These results suggest that prebiotic supplementation may become a strategy to maintain the intestinal barrier integrity and function in the allo-HCT setting.

### 3.8. Lactose-Free Diet

Data from the next-generation sequencing (NGS) of the intestinal microbiome showed that the loss of the microbiota diversity after allo-HCT was associated with increased TRM [59,60,61]. In another study on 3-indoxyl sulfate (3-IS) levels in urine after allo-HCT, it was confirmed that this commensal bacteria metabolite decrease in urine was an indirect marker of decreased microbial diversity and was associated with higher TRM [62]. Much interest was seen in intestinal *Enterococcus* genus domination and its connection with the reduced overall survival and increased risk of aGVHD and GVHD-related mortality. *Enterococcus faecium* requires lactose for optimal growth. Indeed, patients with a lactose-malabsorption genotype had a greater prevalence of *Enterococcus*. In an animal model of allo-HCT, lactase loss in the small intestine over the post-transplantation period was seen, probably due to the intestinal mucosa damage by conditioning. Lactose-free nutrition inhibited *Enterococcus* expansion, alleviated GVHD and reduced the post-transplant mortality in the animal model [63]. All this data suggested a strong base for studies with a new alimentary strategy, i.e., lactose-free diet to improve the allo-HCT recipient outcomes.

### 3.9. Fecal Microbiota Transplantation

Fecal microbiota transplantation (FMT) is a procedure of administration of fecal preparation from a healthy donor into a recipient with the intent of repopulating the patient’s microbiome with diverse microorganisms. Fecal microbiota transplantation in patients with various hematological malignancies colonized with antibiotic-resistant bacteria (ARB) led to the complete eradication of ARB in 75% of recipients [64] and 70% in the case when FMT was performed in a study of 10 patients before or after allo-HCT [65]. The ODYSSEE study investigated the use of autologous FMT in 62 acute myeloid leukemia (AML) patients during induction chemotherapy. In this setting, FMT managed to restore the patients’ diverse microbiome, as well as decrease the antibiotic resistance gene carriage and reduce the intestinal inflammation [66]. In the allo-HCT setting, two case series reporting its utility in recurrent *Clostridium difficile* infection in the posttransplant period were published. No serious adverse effects were noted, and a high efficacy was observed [67,68]. Several case series and pilot studies were investigating FMT safety and efficacy in steroid-refractory gastrointestinal aGVHD. No adverse side effects were reported, and the majority of the patients experienced the clinical benefit of various ranges [69,70,71,72,73]. A prospective trial on FMT efficacy in ARB decolonization and GVHD management in 13 patients with GVHD (11 aGVHD and 2 cGVHD) proved FMT to be an important approach in this setting. In 71% of cases, FMT resulted in the decolonization of at least one ARB. In the case of aGVHD, the overall response rate was 57%, including complete remission in 42% of the procedures. Both patients with cGVHD benefited clinically by stabilization or disease control improvement [74]. Fecal microbiota transplantation, in order to reinduce microbial diversity in the posttransplant period, was studied in a pilot study of 13 patients undergoing allo-HCT. The procedure was well-tolerated and resulted in increased microbiome diversity measured with urinary 3-IS and stool 16S ribosomal sequencing [75]. Autologous FMT offered to 14 allo-HCT recipients resulted in the patients’ microbial diversity restoration compared to the controls that did not undergo the treatment [76]. Fecal microbiota transplantation remains a potential tool to restore the microbiome diversity lost in the post-allo-HCT period. Several clinical trials investigating FMT safety and efficacy in the allo-HCT setting are currently ongoing (NCT04059757, NCT03812705, NCT04285424, NCT04280471, NCT03819803 and NCT04269850).

## 4. Conclusions

Many studies support the hypothesis that an injury to the intestinal mucosa, resulting in increased intestinal barrier permeability, along with microbiota diversity loss, is associated with GVHD initiation and propagation and worse allo-HCT recipient outcomes. To date, there is no perfect way to analyze the gut permeability in HCT recipients; the most commonly used permeability assays like SAT fail to distinguish between increased tight junction permeability and epithelial cell damage. Biomarkers like citrulline or REG3 mirrors the mucosal damage extent rather than increased intestinal permeability. Additionally, most data on intestinal barrier damage importance in GVHD development come from in vitro and animal models that differ substantially from clinical settings. Currently, there is no guideline for routine intestinal barrier protection in allo-HCT. New hopes are raised with dietary interventions (prebiotics, vitamin A and lactose-free nutrition) and novel therapeutic strategies like butyrate or indole derivate supplementation and FMT. Well-designed and, ideally, multicenter studies on safety and efficacy will hopefully provide the evidence needed to determine the best practices in intestinal barrier damage prevention.

## Data Availability

Not applicable.
